# Prevalence and psychosocial profile of instagram addiction among Tunisian doctors

**DOI:** 10.1192/j.eurpsy.2023.1408

**Published:** 2023-07-19

**Authors:** N. Messedi, S. Kolsi, E. Fakhfakh, I. Chaari, F. Charfeddine, L. Aribi, J. Aloulou

**Affiliations:** 1 Psychiatry « B » Department, Hedi Chaker University Hospital Center, Sfax, Tunisia

## Abstract

**Introduction:**

Instagram is the most popular social media platform which is frequently used by population today. Besides, this subject has not been well discussed among doctors.

**Objectives:**

To study the prevalence of addiction to instagram among tunisian doctors and to identify its associated factors.

**Methods:**

This was a cross-sectional descriptive study carried out among doctors (interns, residents and university hospital doctor) during the months of septembre and octobre 2022.

A socio-demographic and clinical characteristics were collected using an online anonymous questionnaire from Google form that we distributed via facebook.

Instagram Addiction Scale (IAS) was used to assess Instagram addiction levels. A score above 37 indicates addiction to instagram.

**Results:**

Our sample included 106 patients. The mean age was 32.32 years (SD=5.66 years) and the sex ratio (M/F) was 0.60. More than half (56.6%) were married and lived with their husband. They were residents in 37% and they were using psychoactive substances in 42.5%.

All of participants used other socialnetwork, the most used was facebook (63.2%).

IAS : The mean score was 31.18 (SD=11.64). The prevalence of addiction to instagram was 42.5%.

The instagram addiction was significantly correlated with : age (p=0,0001), the female gender (p=0.043), the celibacy (p=0.0001), the number of children (p=0.0001) and the number of siblings (p=0.049). Residents were more likely to develop an addiction to instagram (p=0.0001).

**Conclusions:**

The study highlighted the high level of instagram addiction among tunisian doctors and identified individuals who were at higher risk. Specific interventions were necessary to deal with the problem of addiction.

**Disclosure of Interest:**

None Declared

